# Aging-associated oxidative stress inhibits liver progenitor cell activation in mice

**DOI:** 10.18632/aging.101232

**Published:** 2017-04-29

**Authors:** Yiji Cheng, Xue Wang, Bei Wang, Hong Zhou, Shipeng Dang, Yufang Shi, Li Hao, Qingquan Luo, Min Jin, Qianjun Zhou, Yanyun Zhang

**Affiliations:** 1 Shanghai Institute of Immunology and Shanghai Chest Hospital, Shanghai Jiao Tong University School of Medicine, Shanghai, China; 2 Key Laboratory of Stem Cell Biology, Institute of Health Sciences, Shanghai Institutes for Biological Sciences, Chinese Academy of Sciences and Shanghai Jiao Tong University School of Medicine, Shanghai, China; 3 Clinical Laboratory, The First Affiliated Hospital of Anhui Medical University, Hefei, China

**Keywords:** aging, stem cells, liver regeneration, neutrophil, reactive oxygen species

## Abstract

Recent studies have discovered aging-associated changes of adult stem cells in various tissues and organs, which potentially contribute to the organismal aging. However, aging-associated changes of liver progenitor cells (LPCs) remain elusive. Employing young (2-month-old) and old (24-month-old) mice, we found diverse novel alterations in LPC activation during aging. LPCs in young mice could be activated and proliferate upon liver injury, whereas the counterparts in old mice failed to respond and proliferate, leading to the impaired liver regeneration. Surprisingly, isolated LPCs from young and old mice did not exhibit significant difference in their clonogenic and proliferative capacity. Later, we uncovered that the decreased activation and proliferation of LPCs were due to excessive reactive oxygen species produced by neutrophils infiltrated into niche, which was resulted from chemokine production from activated hepatic stellate cells during aging. This study demonstrates aging-associated changes in LPC activation and reveals critical roles for the stem cell niche, including neutrophils and hepatic stellate cells, in the negative regulation of LPCs during aging.

## INTRODUCTION

Organismal aging is a process of functional decline due to histologic and biochemical changes in tissues and organ systems with the passage of time [1]. Like all the other organs, there are structural and functional changes in the liver during aging, including reduction in organ size, increased hepatic dense body compartment, declined capacities in Phase I metabolism of certain drugs, altered expression of a variety of proteins, and diminished hepatobiliary functions [2]. Notably, a decrease in regenerative capacity in aging liver has been observed in old patients who had severe viral and toxic injury [3]. In addition, studies on liver transplantation in human patients showed lower graft and recipient survival if donor was in advanced age [4]. Similar results were also observed after liver transplantation in rats [5]. Therefore, investigating the mechanisms of declined regeneration in liver is critical to understand age-associated hepatic pathologies and diseases.

Decreased tissue regeneration and homeostasis are frequently associated with impaired stem cell function [6], implicating alterations of stem cells within tissues and organs during aging. As recently reported, aging-associated phenotypical and functional variations have been observed for adult stem cells or progenitor cells in various tissues, including epidermis [6], muscle [7], blood [8] and brain [9]. Age-related decrements in stem-cell functionality may occur at different levels, including cell-autonomous dysfunction, altered niche where stem cells reside, systemic milieu and the external environment [6, 7, 9]. Liver is an organ with low turnover in homeostasis, but high regenerative capacity under acute injury [1]. However, little is known about the changes of stem cells within liver responsible for liver regeneration upon liver injury during aging.

Liver progenitor cells (LPCs), also known as ‘oval cells’ [10], are a stem cell population within the liver. LPCs have ovoid nucleus and scant cytoplasm, and express both hepatocytic and cholangiocytic markers, such as α-fetoprotein (AFP), cytokeratin 19 (CK19) and epithelial cell adhesion molecule (EpCAM). Upon massive liver injury, LPCs may be activated to proliferate and migrate into the hepatic lobule where they differentiate into hepatocytes and biliary epithelial cells [11]. LPC expansion occurs in many human liver diseases [12] and experimental animal models [13], and treatment with LPCs could prevent liver injury in rodents [14, 15]. Therefore, LPCs play an important role in maintaining the homeostasis and regeneration of the liver. Characterizing biological properties LPCs during aging will be important to gain insight into age-associated liver pathologies and disease.

According to the ‘free-radical theory’ of aging, endogenous oxidants could be generated in cells and resulted in cumulative damage [16]. Those oxidants, free reactive oxygen species (ROS), including free radicals, oxygen ions and peroxides, are specific signaling molecules regulating biological processes under both physiological and pathophysiological conditions [17]. Within certain extent, the generation of ROS is essential to the maintenance of cellular homeostasis [17]. However, excessive generation of ROS might lead to the damage of various cell components and the activation of specific signaling pathways, which will influence aging and the development of age-related diseases [17].

Neutrophils can be recruited by a variety of cytokines or signals. Neutrophils exert anti-microbial function through phagocytosis (ingestion), release of soluble anti-microbials (including granule proteins) and generation of neutrophil extracellular traps. Neutrophil-derived ROS, including O_2_^−^, hydrogen peroxide (H_2_O_2_), hydroxyl radicals and hypochlorous acid [18], are generated during the process of respiratory burst and are important for neutrophil bactericidal activity [19]. Previous studies have found that spontaneous ROS production from neutrophils may increase with age and represent the different aspect of age-associated immune dysregulation [20].

In this study, taking advantage of a well-established murine LPC activation model, we investigated the alterations in LPC functionality and liver regeneration during aging, and uncovered that the decreased activation and proliferation of LPCs were due to excessive ROS produced by neutrophils infiltrated into niche, which was resulted from chemokine production from activated hepatic stellate cells during aging. Our analyses reveal a new cellular mechanism underlying changes in LPC functionality during aging.

## RESULTS

### Liver progenitor cell activation decreases with age

**Figure 1:**
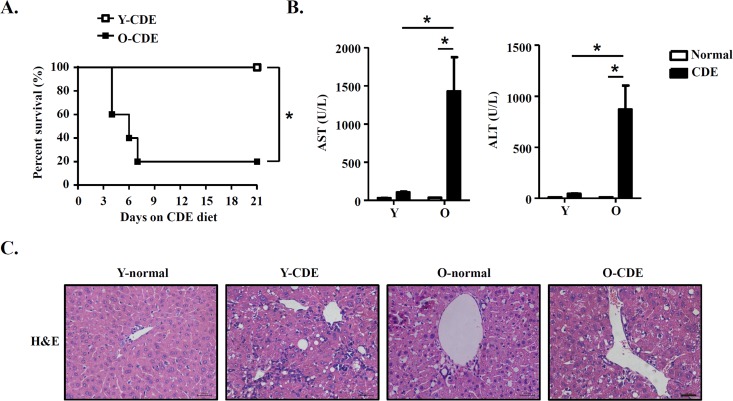
Liver injury in old mice with CDE diet Y/O mice were fed with normal/CDE diet for 3 weeks. (**A**) Cumulative survival rates of Y/O mice with CDE diet were analyzed (n = 5 mice per group). (**B**-**C**) Serum and liver tissues from Y/O mice fed with normal/CDE diet were sampled on day 21. (**B**) Serum levels of AST and ALT were measured. Results are mean ± SEM from three independent experiments (n = 6 mice per group). (**C**) Liver tissues were sectioned for histological examination. Scale bar = 100 μm. Representative images from one experiment out of three are shown. *P < 0.05.

To determine the effects of aging on LPC activation and liver regeneration, we fed mice with modified CDE diet to induce liver damage [21], and examined activation and proliferation of LPCs in young (Y) and old (O) mice. Mice fed with normal diet were used as control. Following CDE diet, young mice were resistant to CDE diet and survived for the 3-week period of the study, consistent with previous report [21]. In contrast, old mice exhibited a high sensitivity to CDE diet, with approximately 80% of them died (Fig. 1A). These CDE diet-fed O-mice (O-CDE mice) showed a dramatic increase in serum aspartate aminotransferase (AST) and alanine aminotransferase (ALT) levels at day 21 compared with CDE diet-fed Y-mice (Y-CDE mice) (Fig. 1B). Histology showed that ductular reactions (formation of ductular structures) were induced in Y-CDE mice as reported, whereas not so obvious in O-CDE mice (Fig. 1C). These results suggest that O-mice have impaired capacity in liver regeneration in response to CDE diet.

**Figure 2:**
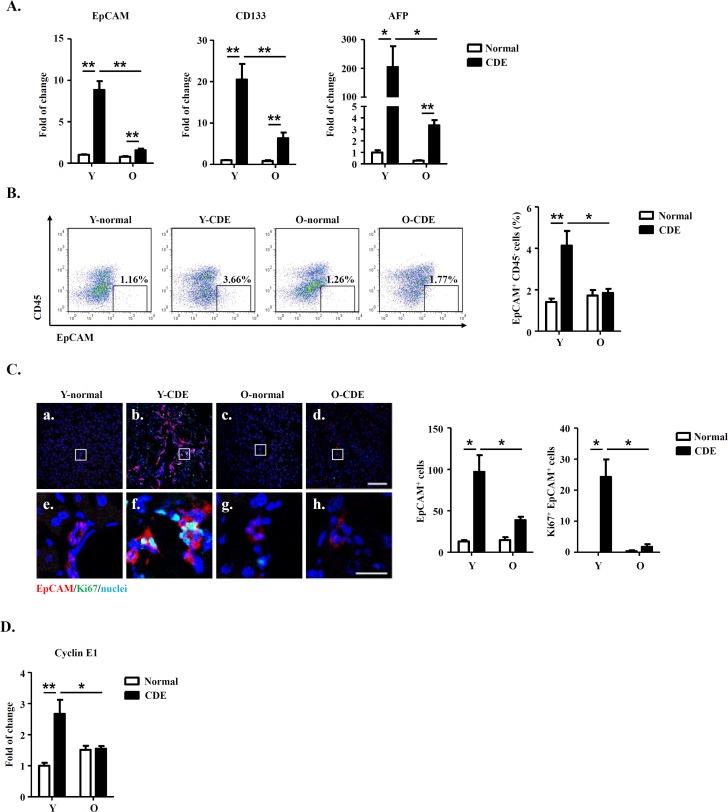
LPC activation and liver regeneration are impaired in old mice Y/O mice were fed with normal/CDE diet for 3 weeks. At day 21, liver tissues from Y/O mice were collected and analyzed. (**A**) mRNA levels of EpCAM, CD133 and AFP in livers were measured by Q-PCR. Results are mean ± SEM from three independent experiments (n > 6 mice per group). (**B**) EpCAM^+^CD45^−^ cells in NPCs from Y/O mice with normal/CDE diet were analyzed by FCM. Percentage of EpCAM^+^CD45^−^ cells was quantified. Results are mean ± SEM from three independent experiments (n > 3 mice per group). (**C**) EpCAM(red)/Ki67(green)/DAPI(blue) staining of liver tissues. a-d, scale bar = 100 μm; e-h, scale bar = 20 μm. Numbers of EpCAM^+^ and EpCAM^+^Ki67^+^ cells were quantified. Results are mean ± SEM from three independent experiments (n > 3 mice per group). (**D**) mRNA level of cyclin E1 in EpCAM^+^CD45^−^ cells from Y/O mice with normal/CDE diet was measured by Q-PCR. Results are mean ± SEM from three independent experiments (n > 6 mice per group). *P < 0.05, **P < 0.01.

To test this possibility, we first measured mRNA expressions of LPC markers (including EpCAM, CD133 and AFP) in livers of normal/CDE diet-fed Y/O mice. As compared to normal mice, expressions of EpCAM, CD133 and AFP were increased in livers of Y-CDE mice (Fig. 2A). Of note, O-CDE liver had marked lower levels of EpCAM, CD133 and AFP than Y-CDE liver (Fig. 2A). Furthermore, FCM analysis demonstrated that percentages of EpCAM^+^CD45^−^ LPC cells in NPCs were lower in O-CDE mice than those in Y-CDE mice (Fig. 2B).

IF staining demonstrated that after CDE diet feeding, O-CDE mice had significantly lower numbers of EpCAM^+^ and Ki67^+^EpCAM^+^ LPCs compared with Y-CDE mice, suggesting decreased level of LPC proliferation in O-CDE mice (Fig. 2C). Next, we sorted EpCAM^+^CD45^−^ LPCs to test the expressions of cyclin A2/B1/D1/E1. As expected, expression of cyclin E1 was increased in LPCs from Y-CDE mice compared with that of Y-normal mice, indicating stronger LPC proliferation in Y-CDE mice. In contrast, O-CDE mice did not show elevated expression of cyclin E1, which was consistent with lower levels of Ki67-positive cells in the O-CDE mice (Fig. 2D). Taken together, these results indicate that LPC activation and proliferation decrease with age.

### LPCs retain functional capacity during aging

**Figure 3:**
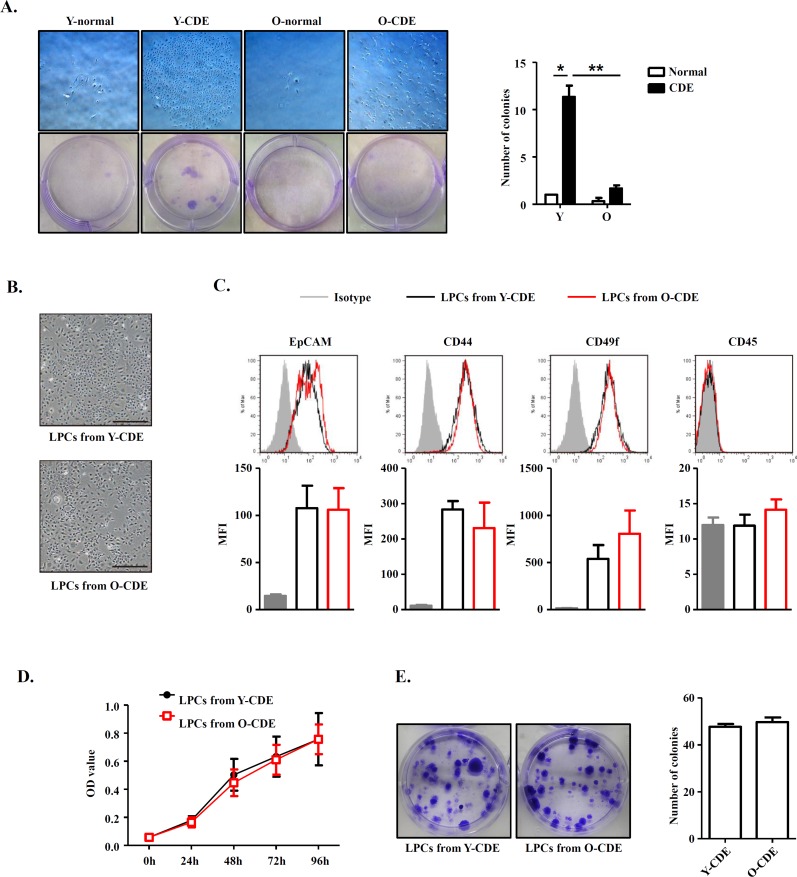
Comparison between LPCs isolated from Y/O mice (**A**) Clonogenic colony-forming assay of freshly isolated EpCAM^+^CD45^−^ cells from Y/O mice with normal/CDE diet. Pictured are wells of each condition. Scale bar = 500 μm. Colony number was quantified. Results are mean ± SEM from three independent experiments. (**B**) Morphology of cultured LPC lines from Y/O-CDE mice. Scale bar = 500 μm. (**C**) LPC lines from Y/O-CDE mice were analyzed for the indicated markers by FCM. Fluorescence intensities of markers were analyzed. MFI indicates mean fluorescence intensity. (**D**) Proliferation of LPC lines *in vitro* culture were analyzed by CCK-8 assay. Results are mean ± SEM from three independent experiments. (**E**) Clonogenic colony-forming assay of LPC lines from Y/O-CDE mice. Pictured are wells of each condition. Colony number was quantified. Results are mean ± SEM from three independent experiments. *P < 0.05, **P < 0.01.

To dissect if the decrease of LPC activation and proliferation in old mice is cell-intrinsic or extrinsic, we next determined the functional capacity of freshly isolated EpCAM^+^CD45^−^ LPCs in Y/O-CDE mice. We plated LPCs isolated from Y/O mice in equal numbers to assess their clonogenic capacity. Primary LPCs from Y-CDE mice produced significantly more colonies than O-CDE mice. Furthermore, the colony size of LPCs from Y-CDE was larger than that of O-CDE (Fig. 3A).

To further assess the proliferative capabilities of LPCs, we established LPC lines from Y/O-CDE mice. In contrast to freshly isolated LPCs, LPC lines showed similar morphology, regardless of their origins, young or old mice (Fig. 3B). In addition, both LPC lines displayed similar phenotypes, as EpCAM^+^CD44^+^CD49f^+^CD45^−^ (Fig. 3C). Furthermore, both Y/O-LPC lines exhibited comparable rate of proliferation (Fig. 3D) and clonogenic capacity (Fig. 3E). Altogether, these results demonstrate that although primary LPCs freshly isolated from old mice have reduced capacity to undergo proliferation *in vivo*, LPC cell lines derived from old mice, which had been passaged for 5 to 15 passages, re-acquired the potent clonogenic and proliferative capacity, indicating the notion that the reduced activation and proliferation capacity might result from alteration of stem cell niche in old mice, although we could not definitively rule out the possibility of an intrinsic defect.

### Increased infiltration of neutrophils in livers of old mice

**Figure 4:**
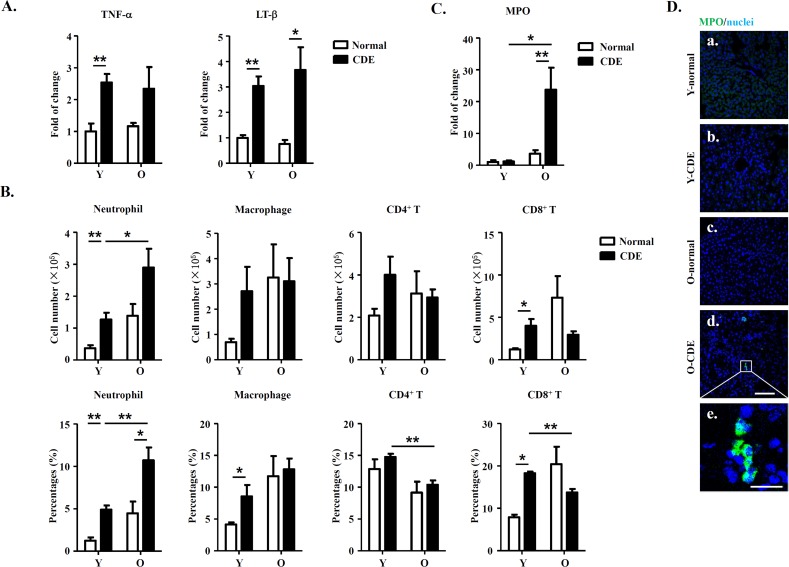
Increased neutrophil infiltration in livers of O-CDE mice (**A**) mRNA levels of cytokines in livers were measured by Q-PCR. Results are mean ± SEM from three independent experiments (n > 4 mice per group). (**B**) Quantification of liver-infiltrating leukocytes from Y/O mice with normal/CDE diet by FCM: neutrophils (CD11b^+^Gr-1^high^), macrophages (CD11b^+^Gr-1^low^), CD4^+^ T cells (CD3^+^CD4^+^) and CD8^+^ T cells (CD3^+^CD8^+^). Results are mean ± SEM from three independent experiments (n > 4 mice per group). (**C**) mRNA level of MPO in livers was measured by Q-PCR. Results are mean ± SEM from three independent experiments (n > 4 mice per group). (**D**) MPO(green)/DAPI(blue) staining of liver tissues from Y/O mice with normal/CDE diet. a-d, scale bar = 100 μm; e, scale bar = 20 μm. *P < 0.05, **P < 0.01.

Microenvironments have been shown to play essential roles in regulating an array of biological events. Since previous studies have demonstrated that immune cells and cytokines, such as T cells, macrophages, and proinflammatory cytokines (TNF-α and LT-β) play important roles in regulating the activation of LPCs in murine CDE model [30], we then determined the expression of TNF-α and LT-β in livers from O-CDE mice using real-time PCR. Of note, we did observe a slight increase for expression of TNF-α and LT-β in CDE mice compared to control normal mice, but no significant difference between Y-CDE and O-CDE mice (Fig. 4A).

Then we focused on the presence of immune cells in liver, and examined the cells by FCM analysis. Our data showed no significant differences in numbers of macrophages (Ly6G^low^CD11b^+^) or T cells (CD4^+^/CD8^+^) between Y-CDE and O-CDE mice. Strikingly, neutrophils (Ly6G^high^CD11b^+^) were greatly increased in livers of O-CDE mice compared with those of Y-CDE mice (Fig. 4B). Consistently, Q-PCR and IF staining showed that expression of myeloperoxidase (MPO), a peroxidase enzyme most abundantly expressed in neutrophil granulocytes, was significantly increased in livers from O-CDE mice compared with that of Y-CDE mice (Fig.4 C,D). Together, these results suggest that neutrophils were increased in livers of O-CDE mice.

### Neutrophils mediate the impairment of LPC proliferation in O-CDE mice

**Figure 5:**
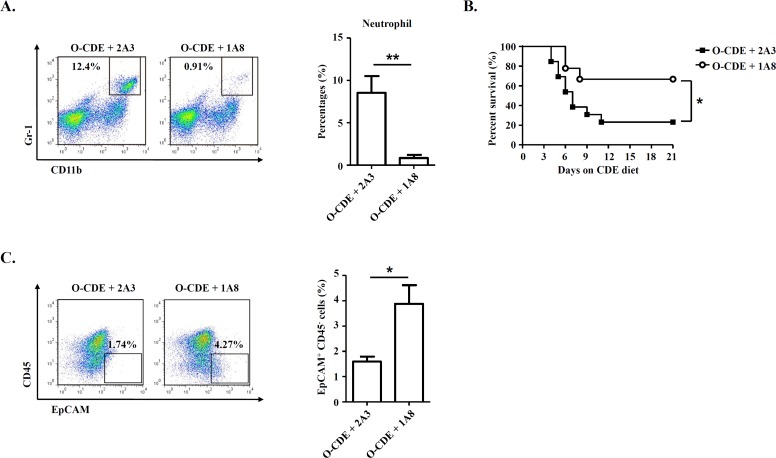
Neutrophils are required for inhibiting LPC proliferation in old mice Old mice with CDE diet were *i.p.* injected with 500 μg of 2A3 (n/a) or 1A8 (anti-mLy6G) mAb every other day to deplete neutrophils. (**A**) Representative FACS scatterplots of liver-infiltrating neutrophils (CD11b^+^Gr-1^high^) in liver tissues from O-CDE mice treated with 2A3 or 1A8 mAb. Percentages of liver-infiltrating neutrophils were quantified. Results are mean ± SEM from three independent experiments (n = 3 mice per group). (**B**) Cumulative survival rates of mice were analyzed. n > 8 mice per group. (**C**) Representative FACS scatterplots and summarized percentages of EpCAM^+^CD45^−^ cells in liver tissues from O-CDE mice treated with 2A3 or 1A8 mAb. Results are mean ± SEM from three independent experiments (n > 5 mice per group). *P < 0.05, **P < 0.01.

To assess the possible impact of infiltrating neutrophils in regulating LPC response in old mice, we administered anti-Ly6G antibody, which could deplete neutrophils [31], to O-CDE mice by *i.p.* injection (Fig. 5A). We found that depletion of neutrophils improved the survival of O-CDE mice considerably (Fig. 5B). Further, depletion of neutrophils restored the LPC response in O-CDE mice as evidenced by the increased percentages of EpCAM^+^CD45^−^ cells in FCM analysis (Fig. 5C). Thus, increased infiltration of neutrophils to the liver of old mice is critical for reducing the capacity of LPCs to proliferate in response to CDE diet.

### Hepatic stellate cells from old mice produce high levels of chemokine CXCL7 to recruit neutrophils

**Figure 6:**
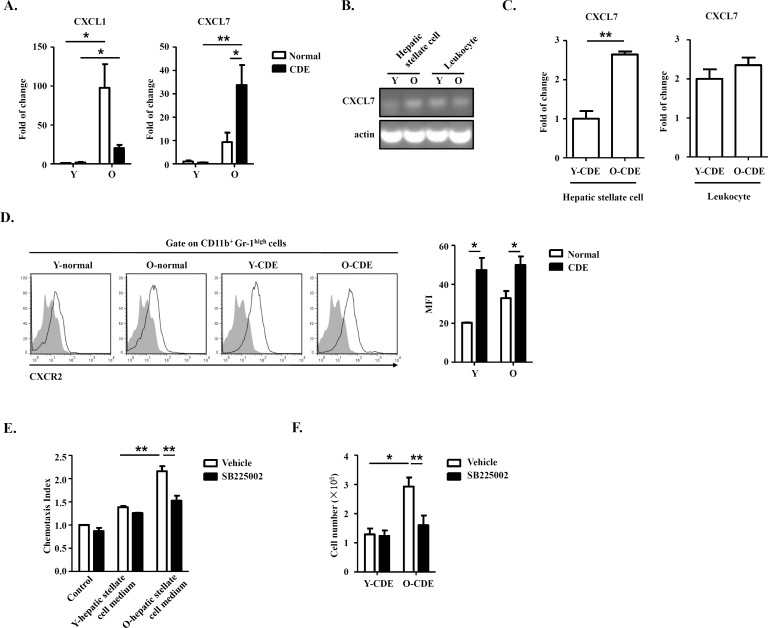
Hepatic stellate cell-derived CXCL7 induced neutrophil infiltration into livers of O-CDE mice (**A**) mRNA levels of CXCL1/CXCL7 in livers from Y/O mice with normal/CDE diet were measured by Q-PCR. Results are mean ± SEM from three independent experiments (n = 3 mice per group). (**B**) PCR analysis of CXCL7 expression in hepatic stellate cells and leukocytes isolated from livers of Y/O-CDE mice. (**C**) mRNA level of CXCL7 in hepatic stellate cells and leukocytes isolated from Y/O-CDE mice with CDE diet were measured by Q-PCR. Results are mean ± SEM from three independent experiments (n = 3 mice per group). (**D**) Levels of CXCR2 expression on neutrophils derived from livers were analyzed by FCM. Fluorescence intensity of CXCR2 was analyzed. Results are mean ± SEM from three independent experiments (n > 3 mice per group). (**E**) Mobility of neutrophils in response to conditioned medium from Y/O-CDE hepatic stellate cells was analyzed in the absence or presence of SB225002 (100 nM). Numbers of migrated neutrophils were determined. Results are mean ± SEM from three independent experiments (n = 3 per group). *P < 0.05, **P < 0.01. (**F**) Quantification of liver-infiltrating neutrophils (CD11b^+^Gr-1^high^) from Y/O mice with normal/CDE diet treated with SB225002 (2 mg/kg) or control by FCM. Results are mean ± SEM from three independent experiments (n > 4 mice per group).

As expressions of chemokines CXCL1 [32] and CXCL7 [33] were reported to be associated with migration of neutrophils in mice, so we tested the expression of CXCL1/CXCL7 in livers in Y/O mice with normal/CDE diet. Q-PCR results revealed that mRNA levels of CXCL1/CXCL7 were upregulated in livers of O-CDE mice compared with those of Y-CDE (Fig. 6A), especially CXCL7, suggesting CXCL7 might be a crucial factor inducing neutrophil infiltration. To investigate from which type of cells was CXCL7 derived, we isolated hepatic stellate cells and leukocytes from liver, the cell populations in liver reported to express chemokines [34, 35]. Gene expression analysis demonstrated that both hepatic stellate cells and leukocytes expressed CXCL7 (Fig. 6B). However, expression of CXCL7 in hepatic stellate cells from O-CDE mice was higher than that in Y-CDE mice, suggesting hepatic stellate cell-derived CXCL7 was responsible for neutrophil infiltration in O-CDE mice. Q-PCR analysis also confirmed this (Fig. 6C).

As CXCR2 is the receptor for CXCL7 and are expressed by neutrophils (Fig. 6D), we next investigated whether hepatic stellate cell-derived CXCL7 recruited neutrophils through CXCR2. We isolated and cultured hepatic stellate cells from Y/O-CDE mice, and conditioned media were collected. *In vitro* chemotaxis assay was conducted to assess the neutrophil migration induced with media from hepatic stellate cells, and data demonstrated that the medium from O-CDE hepatic stellate cells possessed stronger chemotaxis ability on neutrophils. By contrast, neutrophils pretreated with CXCR2 antagonist SB225002 was deprived of migration capability (Fig. 6E). In addition, SB225002 treatment *in vivo* significantly decreased the number of neutrophils (Ly6G^high^CD11b^+^) in the livers of O-CDE mice (Fig. 6F). These data combined show that hepatic stellate cells are responsible for recruitment of neutrophils by producing CXCL7, working through CXCR2 on neutrophils.

### ROS derived from infiltrating neutrophils cause DNA damage to LPCs

**Figure 7:**
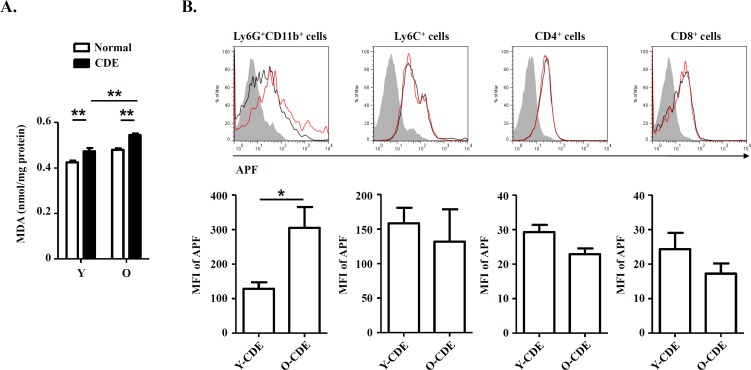
Oxidative stress in livers of O-CDE mice (**A**) Hepatic lipid peroxidation was measured by MDA assay. Results are expressed as nmol MDA/mg liver protein. Results are mean ± SEM from three independent experiments (n > 6 mice per group). (**B**) Level of ROS as assessed by APF fluorescence in leukocytes isolated from Y/O-CDE mice. Results are mean ± SEM from three independent experiments (n = 4 mice per group). *P < 0.05, **P < 0.01.

Studies on primary biliary cirrhosis have already demonstrated that infiltrating MPO^+^ or CD68^+^ inflammatory cells, mainly neutrophils or macrophages, participated in bile duct damage through nitric oxide and ROS [36]. We reasoned that ROS production might contribute to changes of LPC niches in livers of O-CDE mice. To test it, first we measured the oxidative stress in liver samples from normal/CDE diet-fed Y/O mice using MDA, a marker of oxidative stress, as a readout, and we found that MDA level in O-CDE mice was significantly higher than that in Y-CDE mice (Fig. 7A), indicating higher level of ROS in O-CDE mice.

**Figure 8:**
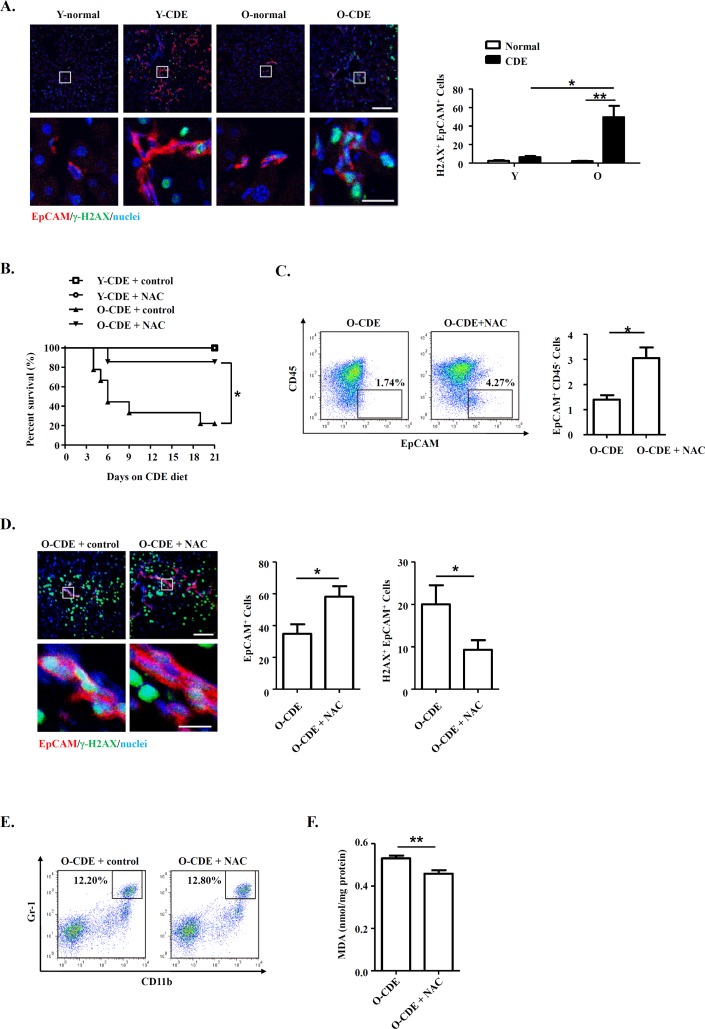
Neutrophils inhibit LPC response through oxidative stress and induce DNA damage during aging (**A**) EpCAM(red)/γ-H2A.X(green)/DAPI(blue) staining of liver tissues from Y/O mice with normal/CDE diet. Numbers of EpCAM^+^γ-H2A.X^+^ cells were quantified. Results are mean ± SEM from three independent experiments (n > 3 mice per group). (**B**) Old mice with CDE diet were subjected to NAC drinking (1 g/L). Cumulative survival rates of mice were analyzed. n > 5 mice per group. (**C**) Representative FACS scatterplots and summarized percentages of EpCAM^+^CD45^−^ cells in liver tissues from O-CDE mice treated with NAC drinking. Results are mean ± SEM from three independent experiments (n > 3 mice per group). (**D**) EpCAM(red)/γ-H2A.X(green)/DAPI(blue) staining of liver tissues from O-CDE mice treated with NAC drinking. Numbers of EpCAM^+^γ-H2A.X^+^ cells were quantified. Results are mean ± SEM from three independent experiments (n > 6 mice per group). (**E**) Representative FACS scatterplots of liver-infiltrating neutrophils (CD11b^+^Gr-1^high^) in liver tissues from O-CDE mice treated with NAC drinking. (**F**) Hepatic lipid peroxidation was measured by MDA assay. Results are mean ± SEM from three independent experiments (n > 6 mice per group). *P < 0.05, **P < 0.01.

It has been proposed that free radicals promote aging [16], and studies have shown that ROS limited the lifespan of hematopoietic stem cells, leading to the exhaustion of the stem cell population [37]. We then tested levels of ROS within liver leukocytes isolated from CDE diet-fed Y/O mice. As expected, levels of ROS in neutrophils from O-CDE mice were higher than those in Y-CDE (Fig. 7B). ROS promotes DNA damage, and the resulted cumulative oxidative DNA damage is considered the key factor in aging and senescence [38]. To understand the mechanism of aging by ROS, we studied the DNA damage in LPCs using the marker, phosphorylation of histone variant H2A.X on serine 139 (γ-H2A.X). As revealed by IF staining, few γ-H2A.X^+^EpCAM^+^ LPCs with DNA double-strand breaks were observed in Y-CDE mice. However, a large percentage of γ-H2A.X^+^EpCAM^+^ LPCs were present in O-CDE mice, suggesting DNA double strand damage in O-CDE LPCs (Fig. 8A).

To further verify the ROS in inducing DNA damage in LPCs from old CDE mice, we administrated antioxidant NAC to the mice, and uncovered the treatment using NAC improved the survival of O-CDE mice (Fig. 8B). Meanwhile, percentages of EpCAM^+^CD45^−^ LPCs were increased in O-CDE mice (Fig. 8C), whereas numbers of γ-H2A.X^+^EpCAM^+^ cells were decreased, as determined by IF staining (Fig. 8D). NAC treatment had no effect on neutrophil infiltration in liver (Fig. 8E). In addition, MDA levels in O-CDE mice were also downregulated after NAC treatment (Fig. 8F). Taken together, we conclude that neutrophils dampen LPC activation through producing excessive ROS in old mice.

## DISCUSSION

Our findings demonstrate that liver regeneration and LPC activation are negatively regulated during aging. Impairment of liver regeneration in old mice might not be resulted from intrinsic changes of LPCs, but from changes of stem cell niche including neutrophils and hepatic stellate cells. Based on our findings, we propose the following model. In old mice, upon CDE-induced liver injury, hepatic stellate cells produce CXCL7 to recruit neutrophils into liver. After neutrophils infiltrate into liver, they are activated and a neutrophil oxidative burst is induced. Then, neutrophil-derived excessive oxidative stress induces DNA double strand damage in LPCs and restricts LPC proliferation, leading to the impairment of liver regeneration. Our findings establish a mechanistic link between LPCs and stem cell niche including neutrophils and hepatic stellate cells, during liver regeneration in old mice.

Our data identify that LPC functionality is negatively regulated by aging. The CDE diet is a well-established tool for inducing the LPC response. In this study, it is very effective for LPC induction by using the CDE model. In addition to the CDE model, we also studied the changes of LPCs in young and old mice fed with 3,5-diethoxycarbonyl-1,4-dihydrocollidine (DDC) diet at the same time. Interestingly, we found that the expressions of LPC related markers, such as Epcam and Afp, were only increased in young DDC-fed mice, suggesting that the activation and proliferation of LPCs in old-DDC mice were decreased compared to that of young-DDC mice which is similar to the results we found in CDE-fed young/old mice (data not shown). Our study, together with previous studies on aging-related changes of other adult stem cells [6], prove that stem cells in various tissues undergo structural or functional changes during organismal aging, linking aging-related changes of stem cells to impairments in tissue regeneration. It is still controversial whether variation in stem cell functionality during aging is resulted from intrinsic or extrinsic factors. In our study, we demonstrate that the aging-related decline in LPC functionality might be regulated by the stem-cell niche surrounding LPCs. Studies on epidermal stem cell have also supported the role of stem-cell niche, which is inflammation in the epidermis, in the regulation of stem-cell functionality during aging [6].

Aging is associated with aberrant inflammatory responses and ROS. In the present study, we observed increased neutrophil infiltration, together with high levels of ROS in livers of old mice. Increased ROS further induced DNA damage in LPCs and inhibited LPC activation in old mice upon liver injury. Although ROS have physiologically essential roles in host defense, overproduction of ROS is highly toxic to host tissues, resulting in severe pathophysiological consequences. In support of our findings, it was found that increased neutrophil-mediated oxidative/nitrosative damage contributed to augmented lung injury in the aged with systemic inflammatory response syndrome [39]. Moreover, MPO, a key enzyme of neutrophil to produce potent oxidants, the expression and activity of which were found to be increased in kidney of rats during aging, contributing to protein oxidation accumulation and tissue damage [40]. The notion that age-related oxidative stress impairs stem cell function was supported by previous study which found that when intracellular ROS levels of hematopoietic stem cells became excessive, they caused stem cell senescence or apoptosis, and finally a failure of stem cell self-renewal [37]. Therefore, the inhibitory role of ROS on stem cell function during aging was confirmed and might be common among different types of stem cells.

Hepatic stellate cells play pivotal roles in liver repair and disease pathogenesis. In our study, we found that neutrophils were mobilized into liver by chemokine CXCL7. PCR analysis demonstrated that expression of CXCL7 was observed in activated hepatic stellate cells. In consistent with our findings, hepatic stellate cells were reported to have the capacity to secrete a group of cytokines that can attract and activate leukocytes, including IL-6, Gro-α (CXCL1), CXCL9, IL-1β [41], CINC [34], and thus promote neutrophilic inflammation in liver [34]. In addition, hepatic stellate cells were reported to exhibit senescence-associated secretory phenotype in mice with obesity, secreting various inflammatory and tumor-promoting factors in the liver, including IL-1β, IL-6 and CXCL1 [41]. Therefore, it is possible that, during aging, hepatic stellate cells adopt aging-related changes and secret chemokine CXCL7 to induce neutrophil infiltration, leading to the negative regulation of LPC response.

Based on our finding that impairment of LPC activation during aging hampered the liver regeneration upon injury, one might assume that impaired LPC function is detrimental to liver function. However, things may not be exactly what they seem. Studies on patients with combined hepatocellular-cholangiocarcinoma have revealed the association between hepatic carcinogenesis and activation of the progenitor compartment [42]. And oval cells isolated from p53 knockout mice are highly tumorigenic, as they can form tumors phenotypically resembling hepatocellular carcinoma after being injected into athymic nude mice [43]. Based on these, it is possible that decreased LPC activation during aging might be a potential tumor-suppressive mechanism within liver along aging. Similar tumor-suppressive mechanisms might also exist in other stem cells during aging, like epidermal stem cells [6].

In conclusion, the current study shows that LPC activation and liver regeneration are dampened in old mice upon liver injury. Furthermore, it is excessive oxidative stress from infiltrated neutrophils that inhibits LPC activation. This work shows that LPC activation and function are negatively regulated during aging, a potential tumor-suppressive mechanism within liver. However, further study is needed to explore the mechanisms involved in the aging-related changes of hepatic stellate cells and the interaction between LPCs and hepatic stellate cells during aging. In addition, how to reach a balance between liver regeneration and tumor suppression should be under consideration in the clinical treatment of liver disease.

## MATERIALS AND METHODS

### Mice

Six- to eight-week-old C57BL/6 mice were obtained from the Shanghai Laboratory Animal Center at the Chinese Academy of Sciences, and maintained under specific pathogen-free conditions at the Chinese Academy of Sciences for up to 2 years. Mice used at 2 and 24 months of age were considered as young or old (Y/O) mice accordingly [7]. All animal experiments were performed in accordance with the guidelines from the Biomedical Research Ethics Committee of Shanghai Institutes for Biological Sciences, Chinese Academy of Sciences. The protocol used was reviewed and approved by the Animal Experiment Administration Committee of the Biomedical Research Ethics Committee of Shanghai Institutes for Biological Sciences, Chinese Academy of Sciences. All efforts were made to minimize the suffering of mice during experiments.

### LPC isolation and culture

Y/O mice were fed with a choline-deficient, ethionine-supplemented (CDE) diet (Trophic Diet, Nantong, Jiangsu, China) to induce LPC activation [21]. In some groups, mice were treated with drinking water containing N-Acetyl Cysteine (NAC, 1 mg/mL; Sigma-Aldrich, St. Louis, MO, USA). In some groups, mice were *i.p.* injected with Ly6G neutralizing Ab (1A8)/control Ab (2A3) (Bio-X-Cell, West Lebanon, NH, USA). In some groups, mice were i.p. injected with 2 mg/kg of CXCR2 antagonist SB225002/control once a day (Sigma-Aldrich). After 3 weeks on the CDE diet, mice were anaesthetized by *i.p.* injection of pentobarbital sodium. Liver nonparenchymal cells (NPCs) and LPCs were then harvested by a modification of a multi-step digestion protocol as previously described [22–24]. NPCs were separated from the hepatocytes by several episodes of low-speed centrifugation (40 × g for 3 min) and were then collected by centrifugation (1500 rpm for 5 min). LPCs were enriched by centrifugation of NPCs through a discontinuous gradient of 20% and 50% PercollTM (Amersham Biosciences, Pittsburgh, PA, USA) in PBS at 1400 × g for 20 min.

Enriched LPCs were then incubated for 30 min on ice with APC-conjugated anti-EpCAM and FITC-conjugated anti-CD45 (eBioscience, San Diego, CA, USA). Then, EpCAM+CD45- cells were isolated by fluorescence-activated cell sorting (FACS) and plated in type I collagen-coated dishes (BD Biosciences, San Jose, CA, USA). The standard culture medium for LPCs is DMEM/F12 (Gibco, Grand Island, NY, USA) supplemented with 10% fetal bovin serum, insulin (1 μg/mL; Sigma-Aldrich), dexamethasone (1 × 10^−7^ mol/L; Sigma-Aldrich), penicillin/streptomycin (1% vol/vol; Invitrogen, Carlsbad, CA, USA), hepatocyte growth factor (HGF, 50 ng/mL; PeproTech, Rocky Hill, NJ, USA), epidermal growth factor (EGF, 20 ng/mL; PeproTech), fibroblast growth factor (FGF, 20 ng/mL; PeproTech) and Insulin-Transferrin-Selenium-Ethanolamine (ITS-X, 1 ×; Invitrogen).

### Histology and Immunofluorescence

Liver samples for histology analysis were fixed in 4% paraformaldehyde and paraffin-embedded. Deparaffinized sections (5-10 μm) were stained with hematoxylin and eosin (H&E; Beyotime, Nantong, Jiangsu, China) as previously described [25]. Immunofluorescence staining of liver sections was also performed as previously described [25]. Confocal images were acquired with LSM 510 microscope (Zeiss, Oberkochen, Germany).

### Flow cytometry (FCM)

For the determination of LPC percentages within liver NPCs, NPCs were incubated for 30 min on ice with the following antibodies: APC-conjugated anti-EpCAM and FITC-conjugated anti-CD45 (eBioscience). For the determination of the subtypes of liver leukocytes, liver leukocytes were incubated for 30 min on ice with the following antibodies: APC/FITC-conjugated anti-Ly6G, PE-conjugated anti-CD11b and FITC/APC-conjugated anti-CD45; FITC/PE-conjugated anti-CD8, PE-conjugated anti-CD4 and APC-conjugated anti-CD45 (eBioscience, San Diego, CA, USA). For the characterization of LPCs cultured in vitro, LPCs were trypsinized and incubated for 30 min on ice with the following antibodies: APC-conjugated anti-EpCAM, PE-conjugated anti-CD44, APC-conjugated anti-CD49f or FITC-conjugated anti-CD45 (eBioscience). For the detection of ROS, liver leukocytes were incubated with 2-[6-(4′-amino)phenoxy-3H-xanthen-3-on9-yl]benzoic acid (APF; Invitrogen, Carlsbad, CA, USA) fluorescence as reported before [26]. FCM measurements were performed with Aria II (BD Biosciences). Data were analyzed using FlowJo software (Tree Star, San Carlos, CA, USA).

### Quantitative real-time PCR (Q-PCR)

Total RNA was extracted from livers at indicated times and was subsequently reverse-transcribed using PrimeScript RT Master Mix (Takara Bio Inc., Otsu, Shiga, Japan). Quantitative real-time polymerase chain reaction was performed using SYBR Green PCR mix (Roche, Basel, Switzerland) on an ABI Prism 7900HT (Applied Biosystems, Carlsbad, CA, US) as previously reported [27]. β-actin was used as an internal control to normalize for difference in the amount of total RNA in each sample. Relative expression of genes was calculated and expressed as 2^−ΔΔCT^. Primers are listed in Table 1.

**Table 1:** Q-PCR primers

Name	Sequence
β-actin forward	5′-TGTCCACCTTCCAGCAGATGT-3′
β-actin reverse	5′-AGCTCAGTAACAGTCCGCCTAGA-3′
CD133 forward	5′-GGAAAAGTTGCTCTGCGAAC-3′
CD133 reverse	5′-TCTCAAGCTGAAAAGCAGCA-3′
EpCAM forward	5′-GATCATCGCTGTCATTGTGG-3′
EpCAM reverse	5′-CACGGCTAGGCATTAAGCTC-3′
AFP forward	5′-CCCTCATCCTCCTGCTACATT-3′
AFP reverse	5′-CGGAACAAACTGGGTAAAGGT-3′
CXCL7 forward	5′-TTCCCATTGAGCATTGTTAT-3′
CXCL7 reverse	5′-TGCTTGACTCCAGGCGATTT-3′
CXCL1 forward	5′-GCTGGCTTCTGACAACACT-3′
CXCL1 reverse	5′-CGCACAACACCCTTCTACT-3′
Cyclin E1 forward	5′-CTCCGACCTTTCAGTCCGC-3′
Cyclin E1 reverse	5′-CACAGTCTTGTCAATCTTGGCA-3′

### In vitro colony assay

For analysis of primary EpCAM^+^CD45^−^ LPCs sorted from NPCs, cells were inoculated at a low density (200 cells per cm2) into 6-well plates coated with type I collagen and cultured in the standard culture medium supplemented with Y27632 (20 μmol/L; Sigma-Aldrich, St. Louis, MO, USA) [24]. After 10-13 days of culture, colonies were stained with crystal violet (Beyotime) and assessed. For cultured LPC lines, cells were inoculated at a low density of 300 cells per well of 6-well plates and subjected to different treatment. After 7 days of culture, colonies were also assessed by crystal violet staining.

### Cell proliferation assays

For proliferation assessed by CCK-8 assay, LPC lines from Y/O mice were cultured in type I collagen-coated 96-well plates at an initial density of 5000 cells per well, in 100 μl of standard culture medium. After 24, 48, 72 and 96 hours, the proliferation of LPCs was determined by using a CCK-8 kit (Dojindo Laboratories, Mashikimachi, kamimashiki gun Kumamoto, Japan).

For proliferation assessed by BrdU staining, LPCs were subjected to different treatment, and BrdU (40 μg/ml) was added 2 hours before the end of the coculture. Immunofluorescent staining of incorporated BrdU was performed according to the BrdU Flow Kit instruction manual (BD Biosciences). Percentages of BrdU^+^ cells were analyzed by FCM.

### Hepatic stellate cell isolation and culture

Hepatic stellate cells from Y/O mice with CDE diet were isolated according to the published protocol [28]. Isolated hepatic stellate cells were plated at a density of 2 × 104 cells/cm^2^. After 48 hours of culture, conditioned medium of hepatic stellate cells were collected for subsequent experiments. And hepatic stellate cells were collected for Q-PCR analysis.

### Neutrophil isolation and chemotaxis assay

Neutrophil were isolated from bone marrow and were incubated with cytochalasin-B (5 μg/ml, 30 min; Sigma-Aldrich), then N-formylmethionyl-leucyl-phenylalanine (fMLP; 10 μM, 1 hour; Sigma-Aldrich) [29]. For chemotaxis assay, freshly isolated neutrophils (1.5 × 10^6^) were placed in the upper chamber of the Transwell inserts (3 μm pore size; Corning Costar, Corning, NY, USA). Inserts were placed in wells containing medium alone (control) or Y/O-hepatic stellate cell-conditioned medium with or without CXCR2 antagonist SB225002 (100 nM). After 120 min, inserts were removed, and migrated neutrophils were collected and counted.

### Western blot

Western blot was performed as previously described [25]. Primary antibody against γ-H2A.X and GAPDH were obtained from Cell Signaling Technology (Danvers, MA, USA).

### Malondialdehyde (MDA) assay

A whole liver homogenate was prepared from frozen liver pieces to measure hepatic lipid peroxidation, using the Lipid Peroxidation (MDA) Assay Kit in accordance with the manufacturer's protocol (Sigma-Aldrich).

### Statistical analysis

Differences were evaluated using Statistical Package for Social Science software (version 16.0, SPSS Inc., Chicago, IL, USA). Mantel-Cox test was performed to estimate cumulative survival rates of mice. Differences between 2 groups were compared using unpaired Student t test or Mann-Whitney U test. Multiple treatment groups were compared within individual experiments by ANOVA. Values of *p* less than 0.05 were considered significant. All data were presented as mean ± SEM.

## Abbreviation

LPCs: liver progenitor cells
ROS: reactive oxygen species
AFP: α-fetoprotein
CK19: cytokeratin 19
EpCAM: epithelial cell adhesion molecule
MDA: malondialdehyde
CDE diet: choline-deficient, ethionine-supplemented diet
ECM: extracellular matrix
